# Hypertensive crisis-induced electrocardiographic changes: a case series

**DOI:** 10.4076/1752-1947-3-7283

**Published:** 2009-08-20

**Authors:** Khalid Abou Farha, André van Vliet, Sjoerd van Marle, Patrick Vrijlandt, Daan Westenbrink

**Affiliations:** 1 PRA-International, Institute for Clinical Pharmacology, Groningen and Zuidlaren, The Netherlands; 2 Cardiology Department, University Medical Centre, Groningen, The Netherlands

## Abstract

**Introduction:**

Myocardial injury is one of the most notorious complications of a hypertensive crisis. Key electrocardiograph signs used to detect cardiac injury such as ST segment changes and cardiac arrhythmias usually indicate acute ongoing end-organ damage. Lack of early signs to predict end-organ damage might lead to a delay in the initiation of therapy and selection of the incorrect therapeutic strategy.

**Case presentation:**

We describe five cases of tall, hyper acute symmetrical T-waves alone or accompanied by other electrocardiograph abnormalities in five healthy participants: three women aged 52, 60 and 62-years and two men aged 49 and 66-years, during a tyramine-monoamine oxidase-inhibitor interaction, phase I clinical trial. T-wave changes appeared early during the course of the hypertensive crisis and were attributed to subendocardial ischemia. The changes were transient and reverted to baseline in parallel with a fall in blood pressure.

**Conclusion:**

Recognition of tall symmetrical T-waves in early phases of hypertensive crisis heralds commencement of myocardial damage. This calls for prompt medical intervention to avoid an impending irreversible myocardial injury. It is our belief that these findings will add new insight into the management of hypertensive crisis and will open avenues of further investigation.

## Introduction

A hypertensive crisis (HC), defined as a rapid and inappropriate intense elevation of blood pressure with or without a risk of rapid damage to target organs such as the heart [1]-[3], is a common presentation to the emergency department and appears without history of hypertensive diseases in 23% of cases [4,5].

Although HC is uncommonly encountered in clinical trial settings, some investigational drug interaction studies, such as tyramine combined with monoamine oxidase inhibitors, might trigger HC [1,3,6]. In this regard, rapid blood pressure (BP) elevation above 180/100 to 110 mmHg [3,5,7] or a sudden increase in systolic BP by more than 60 mmHg above baseline in otherwise cardiovascular healthy patients might be an alarming sign [6].

There are two main subtypes of HC: hypertensive emergency and hypertensive urgency. Clinical distinction between both subtypes is important for risk stratification and initiation of therapy. On the one hand, hypertensive emergency, characterized by life-threatening end-organ damage, requires immediate reduction in BP with parenteral medications. On the other hand, hypertensive urgency, a less aggressive form without evidence of acute target organ damage, requires more conservative therapy aiming to lower BP over a period of hours to days most commonly using oral medications [1,3,5].

One of the tools used in the diagnostic workup of hypertensive crisis is the electrocardiogram (ECG). This might reveal evidence of myocardial ischemia or infarction, typically T-wave inversion and in more severe cases, ST segment displacement [1,7]-[9]. These changes mirror cardiac injury and indicate a hypertensive emergency situation and therefore necessitate prompt medical intervention.

The design of the clinical trial was recommended by the division of Neurology product of the U.S. Food and Drug administration (FDA). The clinical study protocol, amendments and informed consent including statement of willingness (English and Dutch) were reviewed (from legal, ethical and medical points of view) and approved by the independent Ethics Committee, of the foundation "evaluation of Ethics in biomedical research", BEBO Foundation, Assen, the Netherlands. The Committee is accredited by the central committee on research involving human subjects and by the Dutch association of Ethics Committees. The committee is constituted according to the Dutch national act on medical- scientific research in human being, the regulations of the U.S. FDA as laid down in the code of Federal regulations, 21 GFR, part 56 (Institutional Review Board) and the ICH Harmonized Tripartite Guideline E6 on Good Clinical Practice (ICH-GCP). During the review process and prior to approval of the clinical study documents, the members of the committee have taken into consideration the contents of the above mentioned regulations and the declaration of Helsinki (1964) as amended by the 52^nd^ General Assembly, of the World Medical Association (Edinburgh 2000) and the EU Clinical Trial Directive 2001/ 20/ EC.

All subjects enrolled in this trial provided written informed consent before participating in any study-related activity. They were informed about the nature and purpose of the trial, participation and termination conditions, risk and benefits.

The study was conducted in our Clinical facility centre located in the campus of the University Medical Centre, Groningen, the Netherlands.

## Case presentation

The aim of this trial was to evaluate the interaction between tyramine and both selective and non-selective MAO inhibitors. Tyramine was administered in an escalating dose level design of 25 mg ascending up to and including 800 mg during a period of 10 days. This tyramine dose escalation design was, however, discontinued on achieving an increase of more than 30 mmHg in systolic BP (compared with baseline values) on three successive measurements with a period of five minutes between measurements.

As previously mentioned, Prior to study initiation written informed consent was obtained from all participated subjects, 179 healthy male and female volunteers, aged 40-70 year (inclusive). This was followed by their medical history being established; complete physical examination, thorough laboratory investigations and electrocardiograph assessments including exercise electrocardiography. These parameters indicated the healthy non-pathologic status of all participants. At pre-dose time points, baseline assessments of the participants' BP values were determined. After dosing, a regular BP evaluation was performed at five-minute intervals for a period of two hours and thereafter four times hourly for a subsequent period of two hours. Continuous telemetric recording was performed pre-dose to provide baseline reference values, and until at least four hours post-dose.

The trial protocol specified that during the course of the trial, a hypertensive episode of more than 60 mmHg above predetermined baseline values would imply the use of intravenous labetalol, an alpha and beta blocking agent. This was the case in 35 male and female participants. ECGs and/or telemetric recordings during the hypertensive episodes were available from 21 out of 35 participants. In the electrocardiograph records from all 21 patients, clinically important T-wave changes were observed.

The material used for this report was obtained from five participants: three women aged 52-years-old to 62-years-old and two men aged 49 years-old and 66-years-old.

A hypertensive episode with a rapid increase in systolic BP > 60 mmHg above the baseline value has been observed after an oral tyramine dose, as low as 100 mg in the three female participants and as high as 500 mg in the two male participants.

Parallel with the peak BP, four participants, Patients 1 to 4, reported rapid onset of an array of angina symptoms. These were severe headache and neck pain in Patient 1, skipped beats, warm sensation, abdominal palpitation and anxiety in Patient 3, severe headache and chest compression in Patient 2, and throat tightness, palpitation and severe occipital pain in Patient 4. The other male participant, Patient 5, reported no complaints during the paroxysm of BP elevation.

Synchronous with the peak BP levels, telemetric recordings of all five participants showed obvious T-wave changes. The T-waves clearly lost their peculiar asymmetry and became hyper acute, pointed and high amplitude reaching values of ca. 0.7 mV, 0.65 mV, 0.7 mV and 0.9 mV in leads II of, respectively, Patients 2, 1, 3 and 4. These values were 2 to 4 times the values obtained at baseline. In the remaining male patient, Patient 5, where time-matched 12-lead ECG rhythm traces were available, the T-wave amplitudes were 0.5 mV, 1.2 mV, 1.3 mV and 1 mV, respectively, in leads II and V3-V5. Again these values were approximately two times the values seen in the corresponding leads at the baseline time point. All ECG recording electrodes were remounted at identical locations. This avoided inconsistency in voltage tracings obtained at different time points.

**Figure 1:**
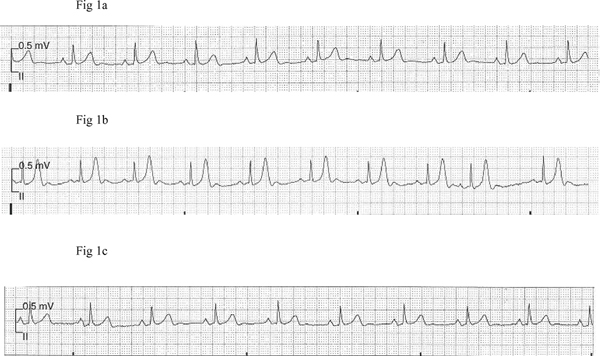
**Telemetric recording obtained (at double standardization, 10 mm = 0**.5 mV) from a female patient depicting tall symmetric T-wave during a paroxysm of hypertensive crisis compared to baseline **(a)** and after normalization of blood pressure **(c)**.

**Figure 2:**
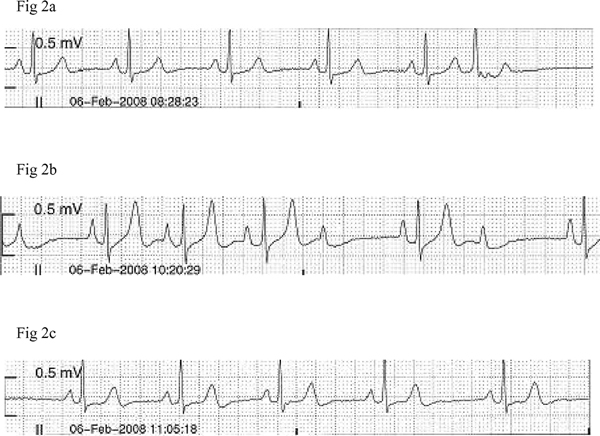
**Telemetric recording obtained (at double standardization, 10 mm = 0**.5 mV) from a female patient, depicting A-V second degree heart block **(b)** during an episode of hypertensive crisis. Notice the tall symmetric T-wave compared to baseline **(a)** and after normalization of blood pressure **(c)**.

**Figure 3:**
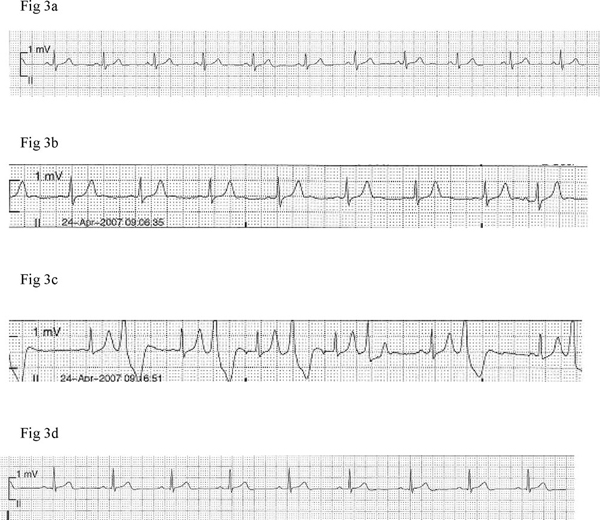
**Telemetric recording obtained (at normal standardization, 10 mm = 1 mV) from a female patient, depicting tall symmetric T-wave seen early in the course of a hypertensive crisis episode **(b)** and in parallel with ventricular bigeminy **(c)****. Recordings a and d demonstrate normal electrical cardiac activities at pre-dose (before tyramine administration) and post blood pressure normalization time points, respectively.

**Figure 4:**
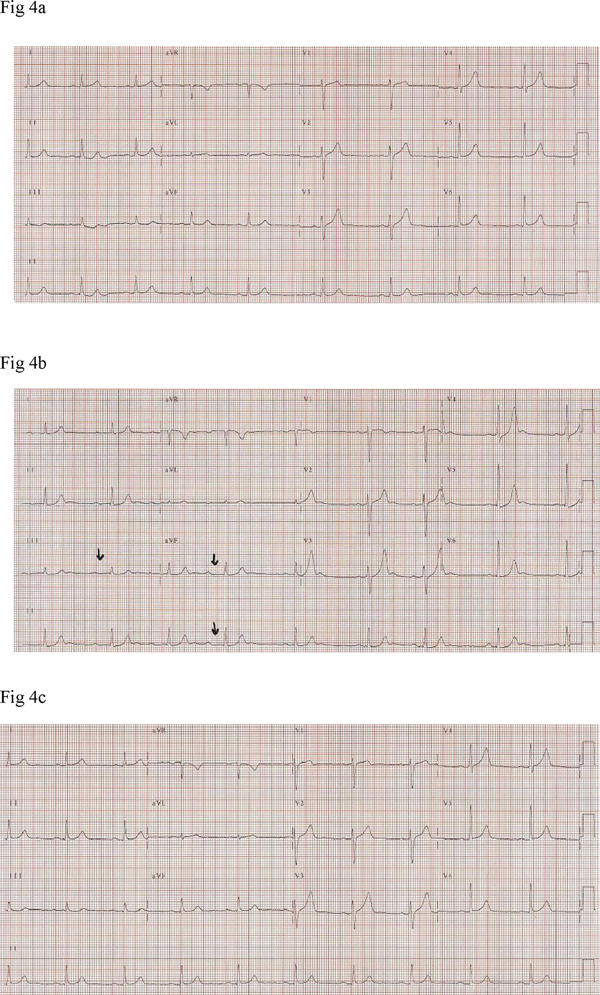
****(a)** Electrocardiogram recordings obtained (at normal standardization, 10 mm = 1 mV) from a male patient, depicting tall symmetric T-wave (seen in V3 to V5) associated with prolonged PR interval as seen in leads II, III and aVF (arrows), during a hypertensive crisis episode**. **(b)** Tracings a and c demonstrate normal electrical cardiac activities at pre-dose (before tyramine administration) and post blood pressure normalization time points, respectively.

**Figure 5:**
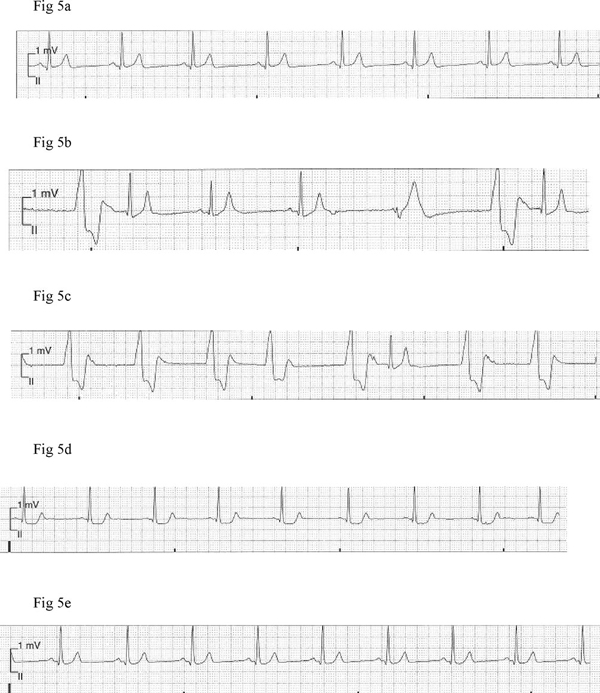
**Telemetric recordings obtained (at normal standardization, 10 mm = 1 mV) from a male patient, depicting tall symmetric T-wave seen in the course of hypertensive crisis **(b)** and in parallel with accelerated idioventricular rhythm **(c)****. ST-segment depression **(d)** is also noted. Figure 5a and 5e demonstrate telemetric recordings obtained at pre-dose (before tyramine administration) and post blood pressure normalization time points, respectively.

In Patient 2, T-wave changes were an isolated event without any associated abnormal telemetric findings (Figure 1). In the remaining four participants T-wave changes were accompanied by other electrocardiograph abnormalities. Patient 1 demonstrated a 2:1 atrioventricular second degree heart block (A-V block) with a heart rate of 60 bpm and narrow QRS complexes but with constant PR and RR intervals between conducted beats (Figure 2). Patient 3 demonstrated ventricular bigeminy (Figure 3), while Patient 5 demonstrated a few sudden cardiac cycles with prolonged PR interval reaching 360 ms, greater than two times the baseline value, with a heart rate of 54 bpm (Figure 4). Patient 4 showed an accelerated idioventricular rhythm (AIVR). This was followed later by evident inferior lead ST-segment depression (Figure 5). All five participants were promptly treated with labetalol, given intravenously as an infusion in doses ranging from 6 to 16.5 mg. Subsequent normalization of BP was followed by clearing of symptoms in the four symptomatic participants and gradual disappearance of associated electrocardiograph abnormalities including the A-V block. In the patient with prolonged PR interval, the PR duration returned to baseline value directly after labetalol treatment. Given the severe ischemic changes observed in the telemetric recording of Patient 4, he was referred to the cardiology care unit of the University Hospital Groningen. Assessment of serum cardiac enzymes revealed a Troponin T level of 0.20 mcg/L, i.e. 20 fold the reference value (<0.01 mcg/L). The patient was subsequently admitted to the cardiology care unit with suspected acute coronary syndrome. Full cardiological investigations were performed including coronary angiography and the patient was discharged three days later after confirmation of a normal cardiac structural and functional profile.

It is noteworthy that inspection of the telemetric recordings of two participants, a female patient with A-V block and the male patient with AIVR, revealed the start of T-wave changes as BP increased by values more than 25 mmHg above baseline.

## Discussion

The extent of clinical and electrocardiographic findings encountered during the trial were unforeseen. However, given the known pharmacological effects of the study medication and the risk of blood pressure changes, the trial was conducted under close and continuous medical observation and monitoring conditions. An adequate and prompt treatment protocol for cases exceeding pre specified safety margin was set up in close collaboration with the intensive care unit of the University Medical Centre Groningen.

We have illustrated the appearance of symmetrical pointed tall T-waves with values of 0.65 mV or higher in lead II of four participants and at least 1 mV in precordial leads of one male participant, early during the course of hypertensive crisis, with an emerging onset at >25 mmHg elevation of systolic BP in two participants, i.e. before reaching values that would normally be denoted as hypertensive crisis. This was associated with angina symptoms in four participants, and followed by characteristic electrocardiograph changes that resolved after labetalol-induced normalization of BP.

Typically, T-waves in healthy patients have non-pointed smooth asymmetric profiles with amplitudes that are age, sex and ECG lead dependent. In healthy male and female patients aged 40 to 60-years, average T-wave amplitudes of 0.23 mV and 0.21 mV, respectively, have been described in lead II of the ECG recording [10].

In leads V3 through V5 of male patients aged 40 to 60-years, reference average T-wave amplitudes of 0.6 mV, 0.54 mV and 0.39 mV have been described [10]. Loss of asymmetrical T-wave profile with increased amplitude is suspicious, especially for patients older than 40 years [11]. In this regard, hyperkalemia, myocardial ischemia and cerebrovascular accidents have all been implicated in the etiology [10].

Our findings can be clarified by the fact that HC causes a sudden increase in the afterload on the heart. This leads to an increase in the myocardial workload and myocardial oxygen demand thereby causing myocardial ischemia [12]. In the very early phases of myocardial ischemia, the first area to be affected is the subendocardium, being farthest from the blood supply [10,12,13]. This leads to a delay in the subendocardial recovery time probably due to activation of the ATP-sensitive K channels [13] that appears electrocardiographically as tall hyper acute T-waves. At the same time, prolonged ventricular sub-endocardial recovery explains the associated cardiac arrhythmias observed in two participants. This point is crucial in planning a therapeutic strategy for HC cases, since tall T-waves appear in the early reversible ischemic phase before myocardial injury occurs. In this context, the clinical significance of T-wave changes is underlined in the HC situation seen in patients with diabetes mellitus who suffer cardiac autonomic neuropathy and lack typical angina symptoms. An appropriate treatment with parenteral first line medications such as labetalol in this early reversible phase might be an end-organ salvage measure.

Interestingly, one male patient demonstrated a prolonged PR interval while, in one female patient, a 2:1 second degree A-V block was observed during the hypertensive episode. The association of the A-V block with HC, symmetrical tall T-waves and the absence of bradycardia indicates ischemic rather than vagally induced heart block as an underlying mechanism. Treating both participants with labetalol led to normalization of BP and was followed by resolution of the A-V block profile and reduction in the associated symptoms in the female patient. The relationship between myocardial ischemia and A-V block including a 2:1 second degree heart block has been previously reported [14].

The central role of myocardial ischemia in the pathogenesis of A-V block seen during HC should, therefore, be considered in the therapeutic approach to non-vagally induced A-V block.

Finally, three points are worth mentioning. First, most of our findings were noted in telemetric records. This facilitates detailed tracing of cardiac electrical activity changes occurring during the hypertensive episodes. Compared to the routine 12-lead ECG tracing, telemetric recordings appear technically a more time sparing procedure, requiring less frequent electrode mounting. In addition, it is a more informative tool, in detecting early cardiac electrical changes associated with HC, given the fact that cardiac electric activity can be displayed on a continuous basis. Therefore, we believe that, in the HC situation, the use of a telemetric recording or bedside monitoring is superior to ECG tracing in risk stratification and thence, offers a timely targeting of a successful treatment strategy. Second, this study is based on incidental observations during a phase I clinical trial. Accordingly, verification of these unforeseen findings with other supplemental investigational tests such as cardiac markers was not scheduled and was not done on any participant except for Patient 4. Further investigations to explore the clinical significance of T-wave changes in HC are, therefore, still warranted. Lastly, the trial gives a sound signal and a clear warning sign to the serious risk of cardiac damage during hypertensive crises. Still worthy of notice, the observed untoward cardiovascular effect resulting from Tyramine administration in supra physiologic escalating doses. This should raise the question whether Tyramine administration in ascending doses could be justified in future clinical trials.

## Conclusions

An asymmetric tall T-wave is an important early ECG myocardial ischemic sign which should not be overlooked in clinical evaluation of patients suffering from drug- or non-drug-related hypertensive crisis. Recognition of T-wave abnormalities should facilitate rapid and appropriate interventions that might abort the cascade of progressive cardiac damage. In the setting of hypertensive crisis, an A-V block associated with an asymmetric tall T-wave, particularly in the absence of bradycardia, should draw attention to the ischemic nature of the disorder which usually requires controlling of BP by parenteral first line medication.

## Consent

Written informed consent was obtained from all participants for publication of this case series. Copies of the written informed consent are available for review by the Editor-in-Chief of this journal.

## Competing interests

The authors declare that they have no competing interests.

## Authors' contributions

KA was involved in evaluation of participants, reported the findings and had primary responsibility for drafting this manuscript. AVV was actively involved in evaluation of the findings and revised this article. SM, DW and PV have revised this article for important intellectual content. All authors read and approved the final manuscript.
